# The Molecular Interaction of Collagen with Cell Receptors for Biological Function

**DOI:** 10.3390/polym14050876

**Published:** 2022-02-23

**Authors:** Jeevithan Elango, Chunyu Hou, Bin Bao, Shujun Wang, José Eduardo Maté Sánchez de Val, Wu Wenhui

**Affiliations:** 1 Department of Marine Bio-Pharmacology, College of Food Science and Technology, Shanghai Ocean University, Shanghai 201306, China; ytxiaoyu@163.com (C.H.); bbao@shou.edu.cn (B.B.); 2 Department of Biomaterials Engineering, Universidad Católica San Antonio de Murcia, 30107 Murcia, Spain; jemate@ucam.edu; 3 Co-Innovation Center of Jiangsu Marine Bio-industry Technology, Jiangsu Ocean University, Lianyungang 222005, China; shujunwang86@163.com

**Keywords:** collagen, receptors, cell signal, biological function

## Abstract

Collagen, an extracellular protein, covers the entire human body and has several important biological functions in normal physiology. Recently, collagen from non-human sources has attracted attention for therapeutic management and biomedical applications. In this regard, both land-based animals such as cow, pig, chicken, camel, and sheep, and marine-based resources such as fish, octopus, starfish, sea-cucumber, and jellyfish are widely used for collagen extraction. The extracted collagen is transformed into collagen peptides, hydrolysates, films, hydrogels, scaffolds, sponges and 3D matrix for food and biomedical applications. In addition, many strategic ideas are continuously emerging to develop innovative advanced collagen biomaterials. For this purpose, it is important to understand the fundamental perception of how collagen communicates with receptors of biological cells to trigger cell signaling pathways. Therefore, this review discloses the molecular interaction of collagen with cell receptor molecules to carry out cellular signaling in biological pathways. By understanding the actual mechanism, this review opens up several new concepts to carry out next level research in collagen biomaterials.

## 1. Introduction

Collagen regulates several intrinsic and extrinsic signaling pathways generally involving regeneration mechanisms. By triggering microenvironment cues, collagen greatly improves the healing process. Several studies have been initiated to explore the potential of collagen in tissue engineering [1,2]. Notably, the potential application of collagen in the regeneration of bone, cartilage, vascular, lungs, nerve, dental, and skin cells has been reported [3]. Our recent studies disclose that collagen biomaterials from marine sources regulate the homeostasis of bone cells such as mesenchymal stem cells, osteoblast lineage cells, osteocytes, marrow macrophages and osteoclasts; dental cells such as periodontal ligament fibroblasts and dental pulp stem cells; and immune cells such as 6T-CEM, T cells and lymphocytes cells [1,4,5]. Empirical evidence reveals that collagen could be used in the form of films, hydrogels, scaffolds, microspheres and bio-inks for tissue engineering applications. Due to the unique characteristics of collagen, researchers are exploring potential sources from which we can obtain superior collagen. Based on the available literature, collagen can be extracted from land-based animals such as buffalo, bovine, calf, chicken, camel, porcine, rat and goat, and marine-based animals such as fish, sea-cucumber, seahorse, sea urchin, sharks, whales, jellyfish, starfish, sponges and bivalves [6,7,8,9,10,11]. Since collagen exists in most internal and external organs, it can be isolated from almost of all parts of animals; however, considering yield, it is most appropriate to use skin, heads, bones, feet, cartilages, tails, fins, scales and gall bladders of animals for collagen extraction. Recent studies have initiated in vitro synthesis of collagen commercially by recombinant DNA technology using vectors [12] to expel the use of animal sources.

Regardless of raw materials, it is important to understand how collagen’s interaction modulates biological cellular activities during development, regeneration and repair to ensure tissue homeostasis. The interaction of collagen with biological cells is mainly facilitated by specific receptors that bi-directionally transmit mechanical signals from inside to outside and vice versa. To support this statement, Barczyk et al. [13] proved that cell surface receptors are well-known key regulators for the direct binding of extracellular matrix proteins with cells. Specifically, receptors such as receptor tyrosine kinases, integrins, immunoglobulin-like receptors and leukocyte receptor complex are shown to use collagens as their potential ligands [14]. Collagen can bind with multiple receptor families to induce a diversity of cellular effects. Though the collagen receptor-mediated cellular response seems to be similar, the molecular interactions between collagen and its receptor are different; for instance, matrix-incorporated collagen fibrils, small collagen fragments and large triple-helical fragments interact differently with cell receptors and mediate unique physiological functions. These research findings highlight the essence of the molecular pattern of collagen in biology.

Additionally, the interaction of collagen with cells is closely related to growth factors and other modulations of the cytoskeleton complex. In general, extracellular signals of collagen often trigger specific cellular cascades pathways such as MAPK, RUNX2, JNK, ERK, STAT, NF-KB, ITAM, FcRγ, etc., to carry out biological reactions. Based on these facts, this review covers the pathways of the signaling interaction of collagen with biological cells in terms of regeneration and other biological applications. Therefore, this review helps biomaterial scientists understand the basic fundamental background of collagen in tissue engineering, which will create a new platform for innovative theories in collagen biomaterial research.

## 2. Cell Receptors for Collagen

As an abundant ECM protein, collagen possibly binds with at least five different groups of receptors in biological cells based on the available data. These are integrins, DDR, Glycoprotein VI, Osteoclast-associated receptor (OSCAR), LAIR-1, and uPARAP/Endo180. It is reported that collagen plays a biological role by binding with one or more of these receptors and activating cellular function.

### 2.1. Integrins

Integrins are well-known cell surface heterodimeric receptors distributed to almost all biological cells, which regulate the development and pathological processes of organs. Integrins constitute a major group of receptors for extracellular matrix components, including collagens. Integrins are widely distributed in organs such as skin, kidney, lungs, muscles, inner ears, eyes, heart, blood vessels, vascular endothelium, heart, gut (Peyer’s patches), lymphatic endothelium, mesenchymal tissue, cartilage and periodontal ligaments; and cells such as macrophage, platelets, fibroblasts, immune cells, bone cells, cancer cells, leucocytes (all types), eosinophils, chondrocytes, embryonic stem cells and so on [15]. Integrins play an essential role in regulating cell signals, migration, survival and differentiation [13,16]. At present, four types of collagen-binding integrins have been identified, namely alpha 1 beta 1 (α1β1), alpha 2 beta 1 (α2β1), alpha 10 beta 1 (α10β1) and alpha 11 beta 1 (α11β1), though there are 24 different types of integrins (formed from 18 α and 8 β subunits) in humans. These four collagen receptor integrins, (α1β1, α2β1, α10β1 and α11β1) are classified under the integrins α1 domain subgroup [17,18,19]. Though the four integrins are reported as collagen-binding receptors, they are expressed in a different location and carry unique signals, for instance, the primary site of integrin expression as follows: fibroblasts and mesenchymal tissues for α1β1 integrin; platelets, epithelium, fibroblasts, and mesenchymal tissues for α2β1 integrin; cartilage and chondrocytes for α10β1 integrin; and periodontal ligaments for α11β1. These integrins transfer the signals in a bidirectional way from outside to inside and vice versa [13]. The reorganization of collagenous matrix by integrins α1β1, α2β1, and α11β1 during collagen contraction is an important process in wound healing [20,21,22]. Among the four types, the signaling mechanism of α1β1 and α2β1 integrins has especially been reported. These integrins bind to both collagen types I and IV; however, their affinities differ: α1β1 has a higher affinity for collagen type IV, while α2β1 preferentially binds to collagen type I [13,23].

Based on the available evidence from the literature, integrin α1β1 was first discovered by Hemler et al. [24] and is mainly located on mesenchymal, immune and epithelial cells, which preferentially bind collagen I, collagen IV, collagen VI, collagen IX, collagen XIII and collagen XVI and also other types of fibrillary collagens [25,26,27] via the MIDAS motif in the α subunit I domain. Integrin α1β1 is commonly expressed in activated lymphocytes, liver, dermis, visceral, kidney, heart, ganglia, microvascular endothelium, and some vascular smooth muscles [28,29,30,31,32]. Collagen binding with the integrin α1β1 receptor regulates the proliferation of living cells, MMP expression and collagen synthesis.

The α2β1 integrin, also known as VLA-2, GPIa-IIa, and CD49b, was first identified as an extracellular matrix receptor for collagens and/or laminins [33,34]. Integrin α2β1 has been reported to be one of the main collagen-binding integrins present in bone, skin and other internal organs that comprise epithelial cells, immune cells, platelets, fibroblasts, chondrocytes and mesenchymal cells. [23,35,36,37]. Among collagens, fibrillar isoforms of collagen I–III, V and XI could preferentially interact with the α2β1 integrin. Not only fibrillar isoforms of collagen, but also the beaded-filament-forming collagen VI, the transmembrane collagen XIII [38], collagen XVI [26] and network-forming collagen IV [39] are also recognized by integrin α2β1. The interaction of collagen with integrin α2β1 is synchronized by the collagen sequence GFOGER [40,41,42]. It was reported that the interaction of collagens such as type I, II and XI with platelet integrin α2β1 is materialized by the GFOGER motif even without stimulators [43].

Integrin α10β1 is a primary receptor of collagen type II that was first identified on chondrocytes in 1998 [44]. Since cartilage is a major site of collagen type II, integrin α10β1 is most abundant in cartilage tissues of the vertebrae, joints, ribs, bronchi and trachea, and thus the expression pattern is unique compared to other integrins. Later, it was identified that integrin α10β1 also binds with other types of collagen-like collagens I, IV and VI. It is expressed on cardiac cells, chondrocytes, perichondrium, endosteum, bone lining cells, fascia lining skeletal muscle fibers and periosteum.

Integrin α11β1 was identified on human fetal myotubes in 1995 by Gullberg et al. [45]. Like integrin α2β1, integrin α11β1 also binds with fibrillar collagen such as collagen I and XIII. It is expressed in organs such as embryo, muscles, and bone, and cells such as mesenchymal stem cells, myocytes, fibroblasts, bone cells, and monocytes [46]. Aside from the four integrins mentioned above, integrin α6β4 and integrin α5β3 were also recently reported to interact with collagen XVII and Noncollagenous domain (NC1) of type XIX collagen, respectively [47,48].

### 2.2. Receptor Tyrosine Kinases (DDR)

Receptor tyrosine kinase is also known as the discoidin domain receptor (DDR1 and DDR2), which plays an important role in the development and growth of organs and is generally activated by binding with different types of collagens such as collagen I–V [49,50]. Since it regulates organs’ growth, any impairment of DDRs may cause disorders in several organs [51]. The activation of the receptor is generally slow and prolonged by collagen stimulation through binding with tyrosine residue autophosphorylation, followed by receptor internalization [52,53]. Empirical evidence claims that binding of collagen with discoidin-homology domain (DD) induces autophosphorylation of the receptor through upregulated N-cadherin expression and Src signaling [49,51,54,55].

DDR1, a transmembrane tyrosine kinase receptor, is an important collagen receptor for intracellular signals for cell proliferation, survival, homing, and colonization and is expressed in many cells and organs [56]. Triggering phosphorylation of tyrosine kinase domains through dimerization in DDR1 can activate various signaling pathways such as MAPK/ER kinase, P38 kinase JNK or PI3 kinase pathways. Notably, in normal conditions, collagen does not interact with DDR1, though collagen is most abundant in the extracellular matrix. However, DDR1 specifically interacts with collagen in cell proliferation, differentiation, migration, and inflammatory response during chronic diseases such as pulmonary, kidney and vascular infection, and is more specifically overexpressed in tumor state. The physiological function of DDR1 has been regulated by ADAM10-mediated ectodomain shedding [57]. Any imbalance of DDR1 leads to atherosclerosis, fibrosis, temporomandibular joint disorder osteoarthritis, and tumor [58,59,60]. On the other hand, DDR2 is mainly expressed in chondrocytes and is involved in the development of bone and cartilages through increasing matrix metalloproteinase expression [61]. In addition to cartilage, DDR2 is involved in the pathological process of arthritis, wound healing, dwarfism and tumor [62,63,64]. More precisely, DDR1 binds to collagen type I and IV, whereas DDR2 interacts with collagen type I, II, and X.

### 2.3. Immunoglobulin Receptor

Glycoprotein VI (GPVI) is an immunoglobulin-based transmembrane stimulatory receptor that is expressed in megakaryocytes and platelets and specifically binds with Gly-Pro-Hyp amino acid residues of collagen. The non-covalent interaction of GPVI with Fc receptor is attained by the presence of positively charged arginine in the transmembrane region. Additionally, the proline-rich motif of GPVI cytosolic tail selectively binds with the Src homology 3 (SH3) domain of the Src family tyrosine kinases Lyn and Fyn [65,66]. Inside-out signaling of GPVI releases stored mediators to activate integrins during thrombus growth, and GPVI signals can be controlled by immunoreceptor tyrosine-based inhibition motif (ITIM)-coupled receptors such as PECAM-1 (CD31) [67]. Studies also claim that binding of Syk to the FcR-γ chain initiates activation of Syk proteins, tyrosine phosphorylation and phospholipase C γ2 (PLCγ2) [67]. GPVI in platelets binds mainly with collagen during the process of blood coagulation [68].

The G6B receptor belongs to the immunoglobulin (Ig) superfamily and is located in the MHC class III region. There are two types of receptor isoforms—G6B-A and G6B-B—with similar N-terminal and varying C-terminal cytoplasmic tails. This receptor binds with SHP-1 and SHP-2 through phosphorylation of immunoreceptor tyrosine-based inhibitory motifs (ITIMs) in its cytoplasmic tail. This receptor accelerates SHP-1 and SHP-2 through ITIMs in its cytoplasmic domain in order to inhibit signaling pathways. G6B is articulated on platelets and megakaryocytes, and functions as a negative regulator of platelet function [69].

Human osteoclast-associated receptor (OSCAR) is another collagen receptor that belongs to the immunoglobulin (Ig) superfamily. This receptor is expressed in a wide range of myeloid cells and is specifically involved in osteoclast growth induction for bone resorption. The level of OSCAR expression is higher during osteoclastogenesis towards bone resorption, which could be achieved through triggering leukocyte receptor complex and FcRγ. Therefore, OSCAR is mainly essential for the differentiation of osteoclast, since it acts as a vital co-stimulatory receptor for osteoclast function and formation [70,71,72,73]. Collagen binding to OSCAR leads to conscription of immunoreceptor tyrosine-based activation motifs (ITAM) containing FcRγ chains. This process further activates the downstream effect of calcium signaling, which is essentially important for the activation of an osteoclastogenic transcription factor such as the nuclear factor of activated T-cells (NFAT) c1.

### 2.4. Leukocyte Receptor Complex (LRC)

LRC is a typical group of receptors primarily expressed in immune cells and plays a diverse role in immune functions such as autoimmunity, antiviral immunity, and graft tolerance [74,75]. The stimulatory receptors contain short cytoplasmic tails and produce positive cues through ITAM of adapter proteins such as DAP10, DAP12 and FcRγ, whereas the inhibitory receptors act through immunoreceptor tyrosine-based inhibitory motifs (ITIM) with long cytoplasmic tails. Interestingly, both stimulatory and inhibitory receptors of LRC could efficiently bind with collagen. The stimulatory and inhibitory receptors of this family include LAIR-1, OSCAR and GPVI [42,66,70,76]. Among these three receptors, the OSCAR and GPVI were already discussed in the previous section. LAIR-1 is an inhibitory receptor actuated by triple-helical collagen as a ligand through interaction with the collagen-related peptide, triplet (glycine-proline-hydroxyproline GPO)10 [77]. It was first identified in platelets and megakaryocytes [76,77,78]. LAIR-1 is also expressed in osteoclast precursors to downregulate osteoclastogenesis [79]. The phosphorylation of LAIR-1 containing two ITIMs recruits phosphatases SHP-1 and 2 and directly dephosphorylates Zap70, PLCγ and Syk, inhibiting ITAM-mediated stimulation of protein kinases and calcium signaling [76,77].

### 2.5. Other Receptors

#### 2.5.1. Fibronectin

Fibronectin (FBN) is an abundant high MW glycoprotein in the extracellular matrix. FBN has multiple binding domains for several biomolecules such as collagens, proteoglycans and TGF-β; the first isolated domain of FBN, consisting of the 6FNI1–2FNII7–9FNI repeats near the N-terminus, is specific for collagen [80,81]. The FBN has a higher affinity to gelatin (denatured collagen) than collagen, since it plays an essential role in tissue growth and wound repair [82,83]. The binding site of FBN in collagen combined with the cleavage site of matrix metalloproteinase (MMP)-1 and the Arg-Gly-Asp (RGD) motif recognition sited in the 10FNIII of the domain is fundamental for cell adhesion [84,85]. The main function of FBN is to promote cell–basement membrane attachment, macrophage function, fibroblast migration, nerve regeneration, clot stabilization, cell-to-cell adhesion, pathogen (virus, fungus, bacteria, and protozoa) binding to mammalian cells and extracellular matrix and embryogenesis.

#### 2.5.2. Vitronectin

Another glycoprotein of the extracellular matrix that binds with collagen is Vitronectin (VTN), which belongs to the hemopexin family and was first identified in serum, named as serum spreading factor [86]. It is mainly distributed in the extracellular matrix, blood serum, platelets and bone. The main function of VTN is to promote cell proliferation, adhesion, immune defense, hemostasis and fibrinolysis [87,88]. Unlike FBN, VTN has a greater affinity to native fibrillar triple-helical collagens than denatured collagen (gelatin) [89]. Empirical evidence proves the inhibitory effect of VTN towards FBN interaction with collagen, proposing that both proteins (VTN and FBN) have competitive attraction at similar binding sites on collagen. Interestingly, the interaction between VTN and collagen is highly regulated by the glycosylation status of VTN, which controls cell migration and adhesion in the tissues [90].

#### 2.5.3. uPARAP

The urokinase plasminogen activator receptor-associated protein (uPARAP, also known as Endo180) is a multi-domain type I transmembrane glycoprotein that belongs to the mannose receptor family. It has several characteristic protein domains such as a series of 8–10 C-type lectin-like domains, cysteine-rich/ricin B-like domain, N-terminal and a fibronectin type-II domain. uPARAP/Endo180 located on the mesenchymal cell surface plays a major role in collagen internalization and turnover [85,91,92,93]. uPARAP/Endo180 is specifically involved in the primary adhesion of collagen to fibroblasts and speeds up the migration of fibroblasts on a fibrillar collagen matrix [91,92,94]. uPARAP/Endo180 is also highly expressed in bone cells such as osteocytes and osteoblasts at sites of intramembranous and endochondral ossification during development [95].

## 3. Biological Regulation

### 3.1. Integrin-Based Signaling Pathways

Integrin, a major regulatory receptor, contributes to several biological functions of collagen. The molecular interaction of α1β1 integrin with collagen triggers several biological signals such as adapter protein activation, Grb2 recruitment, MAPK activation for cellular proliferation and phosphorylation of focal adhesion kinase (FAK) at Tyr-397 for fibroblast to myofibroblast differentiation in order to activate the Shc-mediated pathway in skin regeneration. In keratinocyte, collagen XVII binding with integrin α6β4 and α1β1 stimulates FAK, PI3K and Rac1 activity as a downstream consequence.

#### 3.1.1. In Proliferation, Cell Survival and Movement

**Figure 1:**
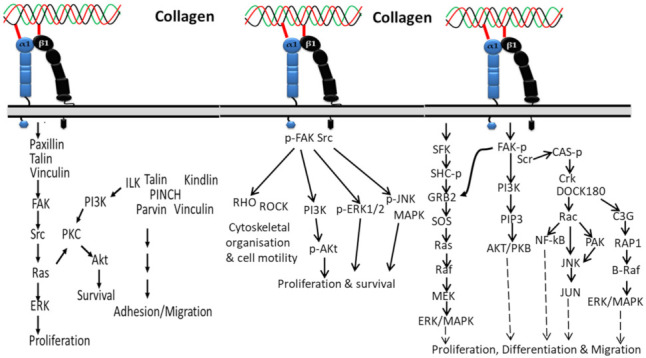
Collagen integrin signals in the normal physiological function of cells.

Integrins have a strong affinity to collagen and transfer several biological signals from the extracellular region to the intracellular region and vice versa [96]. In general, these signals occur through adapter proteins including Src, focal adhesion kinase (FAK), integrin-linked kinase (ILK), kindlin1, paxillin, talin, vinculin, and PINCH of short cytoplasmic tails of integrins (Figure 1). Among the several integrin-associated proteins, kinases such as FAK, ILK, and Src trigger the cellular signal transduction pathways including Akt/PI3K pathways, protein kinase C (PKC) cascades, and MAP kinase pathways (p38, JNK, ERK) [96,97,98]. In addition to signal transfer, integrin-associated protein is also involved in integrin activation [99].

Activation of integrin by collagen phosphorylates FAK/Src pathways, which leads to upregulated cell proliferation and survival via downstream signaling pathways contributing to JNK/MAPK, ERK1/2, PI3K, Akt molecules and transcriptional gene regulation. The activated FAK/Src can also regulate cytoskeletal organization and cell motility in normal and diseased tissue states by mediating Rho/Rho-associated kinase (ROCK) signaling [100].

The two subunits (alpha and beta) of integrin can activate unique cellular activities such as growth, adhesion, differentiation and movement. The intracellular signaling pathway of the alpha subunit of integrin starts by recruiting and activating Src family kinases (SFKs), which additionally recruit FAK through the beta subunit, and the FAK activates signals from phosphatidylinositol 3-kinase (PI3K) to AKT/protein kinase B (PKB) with the help of phosphatidylinositol-3,4,5-trisphosphate(PtdIns(3,4,5)P3), as well as employing Src for focal adhesions. The activated Src is further activated Rac by recruiting the Crk–DOCK180 complex and CAS-paxillin phosphorylation [101]. Then, the activated Rac further triggers NF-κB, Jun amino-terminal kinase (JNK), and p21-activated kinase (PAK) [101,102]. On the other side, the phosphorylated FAK in the beta subunit of integrin can activate ERK/MAPK pathways in two different ways: phosphorylated FAK activates C3G and RAP1 via Crk [103], which leads to the downstream signaling pathway of B-Raf and ERK/MAPK, and the second pathway is involved in activation of the growth factor receptor bound-2 (GRB2) and son-of-sevenless (SOS) complex, which activates Ras, Raf, MEK and ERK/MAPK. In addition, the alpha subunit of integrin is also involved in activation of the ERK/MAPK signaling pathway by directly triggering SFK coupling and SHC phosphorylation, which activates the GRB2-SOS and Ras-Raf complexes [104].

#### 3.1.2. In Cardiac Hypertrophic

**Figure 2:**
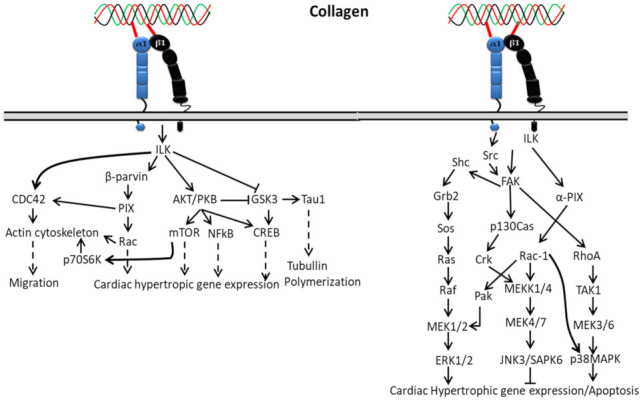
Collagen integrin signals in cardiac hypertrophic gene expression.

Interaction of collagen with α1β1 activates integrin-linked kinase (ILK) through upstream regulators such as phosphatidylinositol 3 kinase (PI3K), PIP3 lipid phosphatase (PTEN) and integrin-linked kinase-associated phosphatase 2C (ILKAP) (Figure 2), which regulate ILK activity by affecting PIP3 binding to the pleckstrin-homology domain of ILK [105,106,107,108]. The activated ILK directly binds to glycogen synthase kinase–3-beta (GSK-3-beta) and protein kinase B (Akt) [106,109], which leads to activation of downstream signaling cascades’ phosphorylation of NF-kappaB, mTOR and CREB during cardiac cell growth. Additionally, the activated ILK is involved in myosin light chain phosphorylation and contributes to Ca^2+^ sensitization of vascular smooth muscle cell contraction [110,111].

In cardiac hypertrophy, the activation of ERK1/2 by collagen-bound integrin directly or indirectly depends on FAK autophosphorylation at Tyr925 through interacting with the Src homology 2 (SH2) domain of Src or Fyn, making a binding region for the signaling complex such as the adapter Grb2 and Ras GTP-exchange factor mSOS. A complex formation of Grb2 with Shc indirectly activates ERK through phosphorylation of FAK at Tyr925 that reframes the cytoskeleton and ERK cascade organization. Shc might also be involved in the initial high-level activation of ERK through a complex formation with Shc/Fyn/Cav-1, though it plays a different role (slow and sustained) in FAK-mediated ERK activation. Hence, based on the above mechanism, FAK can act as an upstream regulator of MAP kinase activity. However, integrins can also stimulate ERK independent of FAK activation through the involvement of PI3K and PKC activation [112].

#### 3.1.3. In Cancer

Empirical evidence confirmed that the intracellular signaling pathways are modulated by increasing integrin (including the α1, α2, α3, α5, α6, and β1 chains) expression on hepatocytes in a fibrotic liver, leading to the development of hepatocellular carcinoma [113,114]. Hence, invasion and growth of hepatocellular carcinoma are highly regulated by integrins α1β1, α2β1 α3β1, α6β1 and α6β4 [99,115,116,117,118].

**Figure 3:**
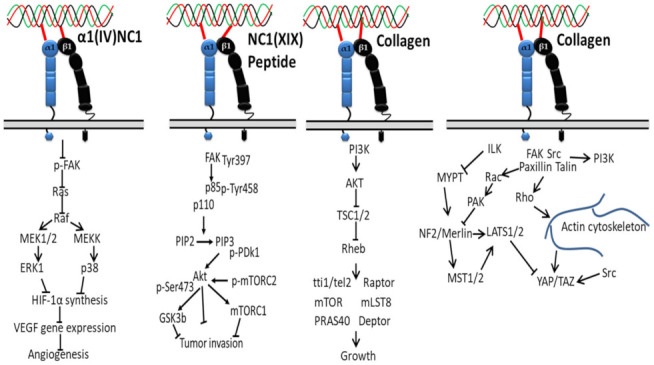
Collagen integrin signals in cancer.

Several noncollagenous domains of type IV collagen (α1(IV)NC1) are reported as novel inhibitors of tumor growth and angiogenesis (Figure 3). For instance, the binding of α1(IV)NC1 with α1β1 integrin inhibits angiogenesis by inhibiting phosphorylation of FAK, Raf/MEK/ERK1/2/p38 MAPK pathways and HIF-1α and VEGF expression, resulting in inhibition of endothelial cells proliferation, migration and tube formation [118].

Another study reported that inhibition of the FAK/PI3K/Akt/mTOR pathway in melanoma cells via subsiding the phosphorylation and activity of major proteins was achieved by the interaction of α1β1/αvβ3 integrin with noncollagenous (NC1) domain of collagen XIX (NC1(XIX)), providing new insight, i.e., anticancer treatments targeting this central signaling pathway in the development of melanoma are promising for the design of new anticancer drugs [47].

Downregulation of TSC1 and TSC2 via activation of AKT and PI3K by collagen-bound integrin indirectly activates mTOR kinase activity through the GTP binding protein Ras homolog enriched in the brain (Rheb), creating a promising cancer therapeutic target [119,120,121]. In another study, collagen type VII-dependent receptor activation of integrin α5β6 downregulates angiogenesis in cutaneous squamous cell carcinomas via expression of p-Smad2, kindlin2 and TGFβ signaling [122].

The proliferation of tumor cells depends on matrix stiffness, less in soft matrix, and is regulated by multiple signaling pathways [123,124,125]. For instance, on a soft surface such as extracellular matrix protein (collagen), the interaction of superfluous collagens to integrin β subunit activates Src family kinases (SFKs) and focal adhesions’ formation by recruiting talin and other cytoskeletal linker proteins, and the signals are transmitted from cytoskeleton to nucleus with the help of the myocardin-related transcription factor (MRTF)/serum response factor (SRF) complex. This system eventually contributes to the tumor cells’ proliferation by accelerating downstream proteins, AP-1 (oncogene c-Jun/c-Fos) via FAK, PI3K, Rac, PAK, MEK, and ERK [126,127]. On a hard surface, the Hippo pathway is involved in the proliferation of tumor cells, consisting of three main components: large tumor suppressor 1/2 (LATS1/2), yes-associated transcriptional regulator/tafazzin (YAP/TAZ) and mammalian Ste20-like kinases 1/2 (MST1/2). In detail, on the stiffer matrix, the ILK-integrin signal inhibits the activity of myosin phosphatase target subunit 1 and suppresses the signaling cascade of Merlin, MST1/2, and LATS1/2 [128], which results in the YAP/TAZ translocation to the nucleus from the cytoplasm [129] and initiated cell proliferation gene (such as cyclin D1 and forkhead box M1) transcription, where they initiate the transcription of genes involved in tumor cell proliferation. In short, collagen–integrin binding recruits focal adhesion signaling molecules, such as FAK, paxillin, Src, Rho, Ras and Rac, ultimately stimulating the progression and contraction of cancer cells [130].

#### 3.1.4. In Epithelial–Mesenchymal Transition

The binding affinity of collagen with different integrin proteins enables cells to express an enormous array of extracellular elements and facilitate unique signaling cascades in response to a changing matrix environment. For instance, the composition of collagen is attributed to a different binding pattern of integrins at the cell surface, contributing the development of epithelial–mesenchymal transition (EMT) under the control of the pericellular environment [131]. Regarding the roles, FAK activation by the b3 integrin subunit, and p38 MAPK pathway, JNK signaling and DAB2 regulation by b1 integrin subunits were reported previously. Furthermore, activation of different types of integrin by collagen triggers significant signaling pathways: a3b1 integrin signals trigger phosphorylation of β-catenin and SMAD2 to promote EMT in a model of lung fibrosis, a5b3 integrins induce EMT by facilitating Src-mediated phosphorylation of TGF-βRII, creating a docking site for ShcA and GRB2 and p38 MAPK pathway, and integrins a5b6 and a5b8 induce the proteolytic release of the latency-associated peptide (LAP), and activate TGF-β at the cell membrane through the protease activity of MMP-14 [131]. Among the different types, type I collagen plays a vital role in EMT induction and metastasis of different carcinomas (lungs, breast and pancreas). In EMT induction, the interaction between collagen and integrin a2b1 triggers intracellular cascade by activating ILK-dependent phosphorylation of IkB, and increasing the abundance of nuclear-localized NF-κB to upregulate the expression of LEF1 and SNAI1. In breast cancer, the pharmacological abolishment of the JNK signal nullifies the collagen-mediated migration and metastasis of tumor cells. A recent report disclosed a ligand-independent role for collagen in stimulating canonical and noncanonical TGF-β signaling [131]. The communication between b1 integrin subunits and pericellular matrix type I collagen is associated with the indirect induction of N-cadherin and the direct suppression of E-cadherin. The above evidence highlights that certain types of matrix proteins interacting with the cell membrane regulate the integrins and thereby control the release of soluble cytokines to induce EMT under the control of the pericellular environment [131].

Overall, the interaction of collagen with integrin is a key regulator in normal and diseased biological processes. The specific binding pattern of collagen with each integrin triggers unique signals for the regulation of various biological mechanisms, for instance, collagen binding with α1β1 activates FAK, ERK, MLCK, p-MLC and E-cadherin in endocytosis; activates ERK/MAPK and PI3K/Akt pathway in osteoblast growth; activates p-α-actinin, vinculin, paxillin, FAK, Shc and Grb2/Erk expression in chondrocytes; activates FAK and ILK signaling pathway to reduce cancer cell–cell adhesion; and inhibits FAK/c-Raf/MEK/ERK1/2/p38 MAPK activation in epithelial cells during antiangiogenic activity and tumor angiogenesis. In the case of α5β3, phosphorylation of p53 at Ser-376 and Ser-378 in PKCα, p53 relocalization (nucleus-cytoplasm), PUMA, Apaf 1, Bax, Bcl-2, Raf, MEK-1, ERK 1/2 and DAPK signals are regulated.

### 3.2. DDR-Based Signaling Pathways

Collagen binds to two types of DDRs including DDR1 (commonly expressed in epithelial cells) and DDR2 (commonly expressed in fibroblasts and mesenchymal cells) spontaneously at the extra/intracellular juxtamembrane domain, N-terminus, tyrosine kinase domain at the C-terminus and transmembrane domain. Collagen binding with the discoidin domain causes conformation changes in DDRs and tyrosine kinase domain phosphorylation, leading to the engagement of ShcA and Nck2 adapter proteins to the cytoplasmic domain of DDRs. Unlike integrin (bidirectional), the ECM cell signal transduction mediated by DDRs is unidirectional.

The action of DDR2 upon collagen II interaction indirectly depends on integrin or cytokines (Interleukin (IL)-1, Toll-like receptor (TLR) ligand) and advanced glycation end products-mediated signaling, resulting in activation of RAS/RAF/MEK/extracellular-regulated kinase (ERK), JNK, MTK, MKKs, p38-MAPK, activator protein 1 (AP-1) (cFos/cJun), E Twenty Six (ETS) factors, Runx2, HIF2α, C/EBPβ and NFκB translocation to nucleus, HIF2α, Elf3, and MMP13 signaling [132,133,134,135].

#### 3.2.1. In Proliferation and Survival

**Figure 4:**
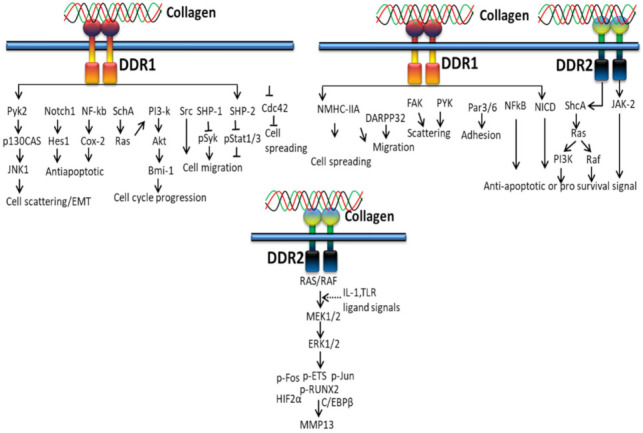
Collagen DDR signals in cell proliferation and survival.

Extracellular fibrillary collagen-bound DDR2 mediates JNK/MAPK and PI3K/Akt signaling pathways to influence gene expression for proliferation and survival [100]. Figure 4 shows the cellular cascades and common intracellular targets organized by the activation of DDR receptor by collagen. DDR1 regulates cell spreading, migration, adhesion and scattering through activating NMHC-IIa, DARPP32, FAK, PYK, Par3/6, NFkB, and NICD, whereas DDR2 regulates anti-apoptotic/pro-survival signals through ShcA-Ras-PI3K, Raf and JAK-2 signals [136].

Collagen stimulation of DDRs intermediates several signaling molecules and adapters such as p85α PI3K, ShcA, STAT1/3/5β, the protein tyrosine kinases (PYK2 and CSK) and the phosphatases (SHP-2 and SHIP-1/2). Not only types of collagen and cells, cell–matrix communication or collagen-independent cell–cell communications also play a major role in triggering different signaling pathways by DDRs. Collagen-bound DDR1 activates JNK, NF-kB, p38, ERK1/2 MAPKs and PI3K/Akt, whereas inactivated DDR1 interacts with E-cadherin supporting cell–cell interactions. DDR1 also works together with other cell receptors such as Frizzled5 and Notch1 to support or alienate collagen-binding integrin signaling pathways linked to cell growth and movement, whereas DDR2 promotes cell migration by interacting with the insulin receptor and, hence, activates MAPKs including p38 and ERK/JAK2 signaling pathway [137].

#### 3.2.2. In Extracellular Matrix Deposition

**Figure 5:**
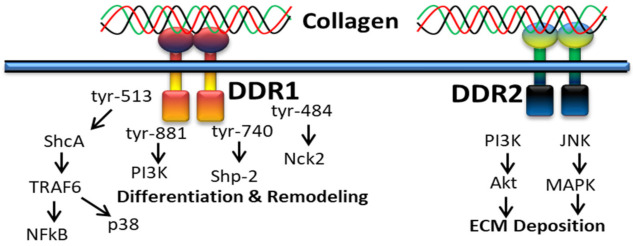
Collagen DDR signals in ECM deposition.

It was reported that collagen-bound DDR2 triggers PI3K/Akt and JNK/MAPK signaling pathways (Figure 5) to upregulate cellular behavior and gene expression for extracellular matrix deposition [100].

DDR1 is mainly involved in cell differentiation and remodeling through various signaling pathways, for instance, alternative splicing of tyrosine-513 of DDR1b is related with the PTB domain of ShcA upon receptor activation [50]. DDR1-mediated phosphorylation of ShcA triggers NFκB pathways and p38 mitogen-activated protein kinase via the TRAF6 complex. In addition, the alternative splicing of tyrosin-484, tyrosine-740 and tyrosin-881 of DDR1 is related to activation of Nck2, Shp-2 and PI3K [138].

#### 3.2.3. In Cancer

DDR1 has been overexpressed in multiple cancers such as non-small-cell lung carcinomas, pancreatic ductal adenocarcinoma, ovarian tumor, breast cancer, gastric cancer, endometrial tumors, glioblastoma, head and neck squamous cell carcinomas, esophageal carcinoma, cholangiocarcinoma, non-small-cell lung cancer, hepatocellular carcinoma, and prostate cancer. Likewise, DDR2 is also overexpressed in acute myelocytic leukemia, thyroid cancer, cholangiocarcinoma, Hodgkin’s lymphoma, breast cancer, and nasopharyngeal carcinoma [130].

**Figure 6:**
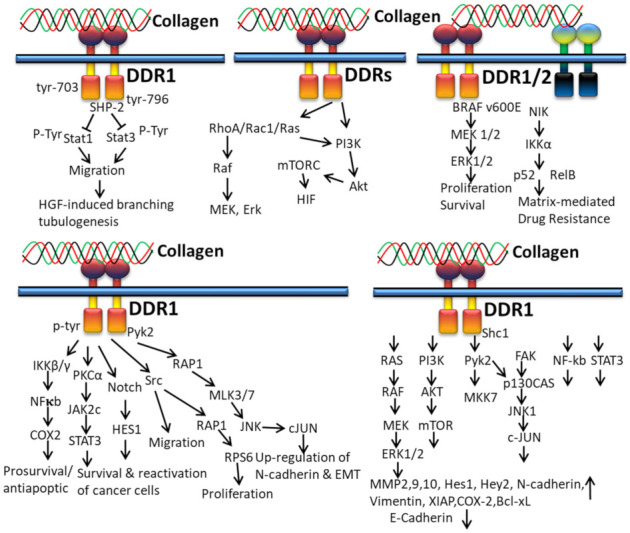
Collagen DDR signals in cancer.

Collagen-bound DDR1 activates the interaction of PTP and SH2-SH2 domains of SHP-2 and the docking sequence in the cytoplasmic domain of DDR1. This interaction between DDR1 and SHP-2 is interrupted by the mutation of tyrosine residues 703 and 796 in DDR1, which subsequently restore collagen-induced cell migration, Stat1/Stat3 activation, and HGF-induced branching tubulogenesis (Figure 6). Ample evidence indicates that collagen-mediated cell migration and Stats activation can be suppressed by the DDR1/SHP-2 complex, which directly interacts with Stat1 and Stat3, leading to dephosphorylation the INF-α-stimulated tyrosine phosphorylation of Stat3 in HeLa cells [139].

It has been reported that collagen from ECM controls cancer cell behavior by regulating invasiveness and mortality of cells [140,141,142,143] and the higher density of type I collagen in tumor microenvironment ECM is related to tumor aggressiveness [144,145]. In addition to integrins, DDRs also play a central role in collagen-induced signaling pathways in cancer cells. The interaction of collagen with DDR1 induces tyrosine phosphorylation and kinase activation of DDR1 and, thus, initiation of multiple downstream signaling pathways: Src kinase activation for proliferation and cell migration [146], activation of proline-rich tyrosine kinase 2 (Pyk2) and N cadherin expression, and Src, Notch, IKK and Pyk2 mediate the RAP1 signaling pathway for regulation of epithelial to mesenchymal transition (EMT) of cancer cells, respectively [54]; regulation of the NFκB-COX2-mediated pathway for chemo-resistance and cell survival; induction of matrix metalloproteinase (MMP) expression for the degradation of extracellular matrix and tumor invasion [147]; and interaction with TM4SF1 for reactivation and survival of breast tumor cells in the metastatic site [148].

In vitro studies disclosed that the action of fibrillary collagen can be regulated by DDR2 in order to accelerate vascular endothelial growth factor (VEGF) expression and lung fibroblasts to undergo myofibroblastic changes through phosphorylation of Akt, p38, ERK1/2 and Smad2. The studies conclude that the activation of myofibroblast and neovessel formation during pulmonary fibrosis can be prevented by targeting the DDR2 signaling pathway [149]. In tumor cells, the interaction of collagen and DDRs triggers PI3K, Akt, mTORC, HIF, Rho A/Rac1/Ras, Raf, MEK, and Erk pathways (Figure 6) [130].

In vitro 3D cell culture demonstrated that the DDR1 enhances chemo-resistance by the STAT3 and NF-kB signaling pathway in Jurkat cells and T47D breast cancer cells [150,151]. The collagen-bound DDR1 upregulates MMP2,9,10, Hes1, Hey2, N-Cadherin, vimentin, XIAP, COX-2 and Bcl-xl, and downregulates E-cadherin expression through RAS/RAF/MEK/ERK1/2, PI3K/AKT/mTOR, Pyk2/MKK7, FAK/p130CAS/JNK1/c-JUN, NF-kb and STAT3 downstream signaling mediators [152].

When collagen interacts with cancer cell DDR1/2, a non-canonical NFκB2 (p52/RelB) resistance pathway is activated. The matrix-mediated drug resistance is achieved by activating NIK, IKKa, and p52, and the MEK1/2 and ERK1/2 regulate the proliferation and survival of melanoma cells [153]

Inhibition of DDR1 resulted in upregulation of E-cadherin and downregulation of N-cadherin and vimentin protein expression, confirming that DDR1 inhibition decreased cell survival and proliferation of prostate cancer cells by downregulating P-DDR1, P-Pyk2, and P-MKK7 levels, which leads to G1 cell cycle arrest and induced cell death by an increase in the Bax/Bcl-2 ratio, depletion of the mitochondrial membrane potential, and reactive oxygen species creation. Further, DDR1 inhibition prevents EMT through the MKK7 and Pyk2 signaling pathways, which cause apoptosis in the prostate tumor cell. Accordingly, DDR1 activates EMT via stimulating the protein expression of N-cadherin and vimentin and phosphorylation of Pyk2 and MKK7 in prostate cancer [154].

#### 3.2.4. In EMT

**Figure 7:**
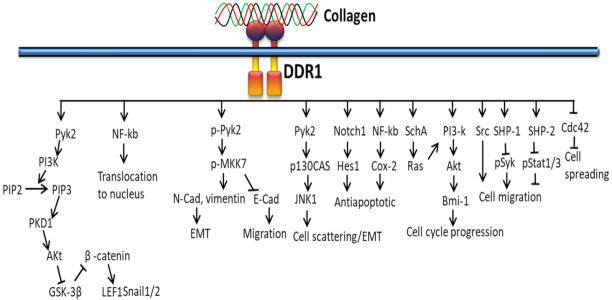
Collagen DDR signals in EMT.

The interaction of collagen and DDR1 promotes EMT via the JNK1–c-Jun pathway with the help of integrins. Along with DDR1, DDR2 also induces EMT by activating NF-κB, LEF-1 transcription factors and other transcription factors (TFs) to upregulate the expression of SNAI1/2 and LEF1 (Figure 7). The activity and stabilization of EMT-associated transcription factors Snail1/2 and LEF-1 are upregulated by collagen-DDR-mediated proline-rich tyrosine kinase 2 (PYK2)–PDK1, ILK, PI3K and the FAK-paxillin pathway. The binding of collagen with DDR disrupts the formation of complexed DDR1-E-cadherin at the cell surface [131].

DDR1 activates ERK 1/2 in mammary epithelial, smooth muscle, transfected embryonic kidney cells, and megakaryocytes [55,155] and suppresses mesangial cells [156]. DDR1 activates JNK in pancreatic cancer cells [54], aromatase transcription through biomechanical signals in adipose stromal cells [157] and PI-3 kinase/Akt signals in normal, cancer cells and embryonic stem cells [158]. In contrast, DDR2 triggers p38 and ERK1/2 (but not JNK) to activate MMP-13 expression in chondrocytes [61,159,160], p38 and JNK (but not ERK1/2) to activate IL-12 production [161], ERK2 in breast cancer cells [162], and p38 MAP kinase or ERK1/2 to activate transcription factor Runx 2 during osteoblast differentiation [51,163].

### 3.3. Collagen/GPVI-Based Signaling Pathways

**Figure 8:**
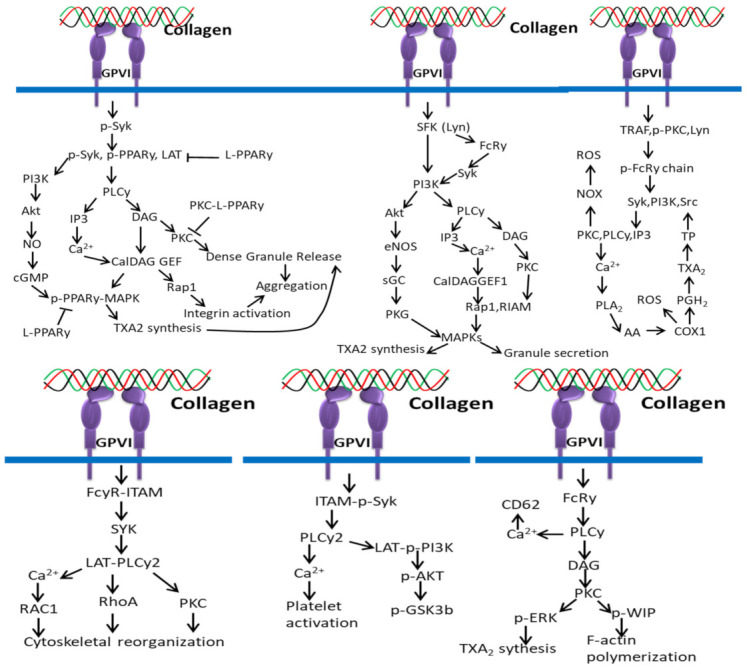
Collagen GPVI signals in platelets activation and ROS production.

The interaction between collagen and GPVI plays an important role in platelet signaling. In platelets, binding of collagen with GPVI recruits PPARγ to interact with the adapter molecule, Syk [164], leading to phosphorylation of linker of activated T cells (LAT) to form a Syk–PPARγ complex (Figure 8). This process is related to subsequent activation of downstream mediators phospholipaseCγ (PLCγ), PI3K, and Akt. Studies claim that the activation of platelets by collagen leads to PPARγ phosphorylation, which interacts with p-ERK and p38 MAPK, leading to granule secretion [165], and the phosphorylated PPARγ downregulates the activation of PKCα in response to PPAR agonists [166,167].

Collagen triggers platelet activation by phosphorylation of downstream signal molecules of the collagen-specific receptor GPVI signal pathway, including ITAM-Syk, PLCy2, and PI3K-Akt-GSK3β, which is inhibited by αIIbβ3-mediated β3-Src signals [168].

More specifically, the interaction of repetitive glycine–proline–hydroxyproline (GPO) motifs of collagen with GPVI dimers triggers subsequent signaling pathways including Src kinases Fyn/Lyn-mediated tyrosine phosphorylation of the FcR γ-chain–ITAMs [67], tyrosine kinase Syk-dependent signaling cascade leading to the formation of a LAT (linker of activated T cells) signalosome, SH2 domain-containing leukocyte protein of 76 kDa (SLP-76), phosphoinositide-3 kinase (PI3K) and phospholipase Cγ2 (PLCγ2) activation [67], which trigger Ca^2+^ mobilization, degranulation, aggregation, and platelet integrin activation. GPVI/FcR γ-chain-mediated signaling in platelets is negatively regulated by immunoreceptor tyrosine-based inhibition motif (ITIM)-containing receptors including CEACAM1, PECAM-1, or G6b [169].

Collagen interaction with GPVI triggers a series of signaling cascades by the release of synthesis of thrombin, and granule-stored mediators [170] such as serotonin, adenosine diphosphate (ADP), platelet-activating factor (PAF), vWF, and TXA2, which activate platelets and further increase the intensity of the entire response, and the entire process is controlled by three classes of PKCs such as atypical (aPKC), classical (cPKC), and novel (nPKC) enzymes [171].

Collagen stimulation is activated by several cellular receptors of the platelets including integrin α2β1 for platelet adhesion to collagen and GPVI for platelet activation. The cytoplasmic domain of GPVI is noncovalently attached to the Fc receptor γ chain (FcRγ) and activates tyrosine phosphorylation of ITAM by SFKs (mainly Lyn and Fyn) with the help of CD148, a receptor-like protein tyrosine phosphatase [172,173]. The phosphorylation of ITAM activates tyrosine kinase Syk, which further phosphorylates downstream targets, such as the Src homology 2 domain-comprising leukocyte phosphoprotein of 76-kDa (SLP-76) and the transmembrane adapter linker for activated T cells (LAT). The activated SLP-76 and LAT induce a signaling complex (SLP-76, LAT, Gads, Bruton tyrosine kinase (Btk), and phospholipase Cγ (PLCγ)) that further supports the synthesis, granule secretion, and integrin activation of thromboxane A2 (TXA2) through activated PLCγ. The interaction of pleckstrin homology (PH) domain of PLCγ2 with the PI3K product phosphatidylinositol 3,4,5-trisphosphate facilitates recruitment of PLCγ2 to the plasma membrane and activation [174,175,176].

ROS production mediated by collagen and GPVI interaction depends on two different pathways such as Syk-dependent and Syk-independent pathways. In the Syk-dependent pathway, the cytoplasmic tail of GPVI-containing TRAF4 and Src family kinase Lyn triggers phosphorylation of ITAM sequences, activating PI3K and Syk, which activates the (PLCγ2)–IP3/PKCs axis, Ca^2+^ mobilization and NOX-mediated ROS production. The higher intracellular Ca^2+^ level activates PLA2 for the production of ROS by COX1 during AA conversion in TXA2. In the Syk-independent pathway, TRAF4 and Lyn activate NOX by interacting with PKC and thrombin-PAR1/PAR4 and release ROS in the extracellular environment. NOX-mediated ROS production is also triggered by the interactions of sCD40L/CD40, ox-LDL/CD36 and TXA2/TP [177].

### 3.4. Collagen-Osteoclast-Associated Receptor (OSCAR)-Based Signaling Pathways

Oscar, a specific collagen receptor to collagen I–III motifs, is involved in the normal development, maintenance and repair of bone. Oscar is widely expressed on human myeloid and osteoclast precursor cells and acts as a positive regulator for collagen-induced osteoclast formation to support bone resorption [70] through the STAT3 pathway. The overexpression of oscar triggers cell adhesion molecules, ICAM-1, and thereby may stimulate the adhesion of monocyte to the endothelium. Collagen activation facilitates the interaction of oscar to immunoreceptor tyrosine-based activation motif (ITAM) with an adapter molecule Fc receptor γ-chain [178] and, thus, regulates STAT signals by ITAM-dependent pathways [179]. In osteoclasts, the expression of Oscar is regulated by STAT3 (positive) and STAT1 (negative) through IFN-γ induction of the MHC class II trans-activator CIITA and the protein inhibitor of activated STAT3 (PIAS3) [180,181,182].

**Figure 9:**
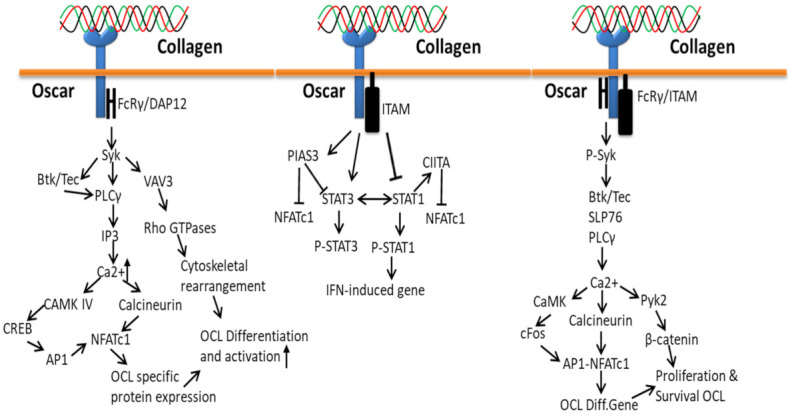
Collagen Oscar signals in bone.

Oscar-FcRγ mediated activation of CAMK IV and calcineurin releases co-stimulatory signals to amplify the induction of NFATc1 during RANK–RANKL interaction in order to support osteoclast activation and maturation (Figure 9). The receptor binding of Oscar triggers phosphorylation of tyrosine residues in FcRγ by members of the Src family, which recruits Syk kinase and stimulates the activity of downstream effectors such as guanine exchange factor VAV3 and phospholipase PLCγ to subsequently activate further targets.

The process of bone development, maintenance and repair starts by the accumulation of osteoclast precursor cells to the collagen-rich bone surfaces from the circulation (blood capillaries) by transendothelial migration that expresses RANKL and collagen I/III [70,183], which triggers the differentiation of precursor cells to the production of multinucleated osteoclast cells [184]. Collagen interaction with Oscar triggers osteoclast differentiation resorption gene (NFATc1) expression through cytoplasmic domain FCRy and Syk phosphorylated downstream signals, which activate PLCy, SLP76, Btk/Tec, Ca^2+^ and calcineurin [185].

Collagen-bound Oscar upregulates the osteoclastogenic effect of RANKL by co-stimulatory signaling pathways through the stimulation of DAP12/FcRγ-Syk-PLCγ signaling cascade that triggers calcium signaling and NFATc1 induction. More specifically, Oscar transduces signals to the immunoreceptor tyrosine-based activation motif in DNAX-activation protein 12 and the Fc receptor common γ subunit and then activates the downstream signals of Syk and phospholipase C γ2 (PLCγ2) [70,186].

### 3.5. Collagen-LAIR1-Based Signaling Pathways

LAIR1 acts as an inhibitory receptor to negatively regulate the osteoclastogenic stimulatory effect of Oscar, even though both receptors belong to the LRC family. Collagen interaction with LAIR-1 (primarily expressed in NK cells) triggers biological signals to maintain the immune tolerance at the maternal–fetal interface by downregulating the activity of NK cells. Collagen-bound LAIR-1 initiates the interaction of SHP-1 to JAK1/2 in NK cells and thereby reduces phosphorylation of STAT1/4 and IFN-γ and TNF-α production. Collagens alone do diminish the expression of natural cytotoxicity receptor NKp30 and perforin production [187], and upregulate inhibitory receptor KIR2DL1 expression on dNK cells. Thereby, collagen has a vital role in reducing cytothe toxicity and activity of NK cells to maintain immune tolerance at the maternal–fetal interface [188].

**Figure 10:**
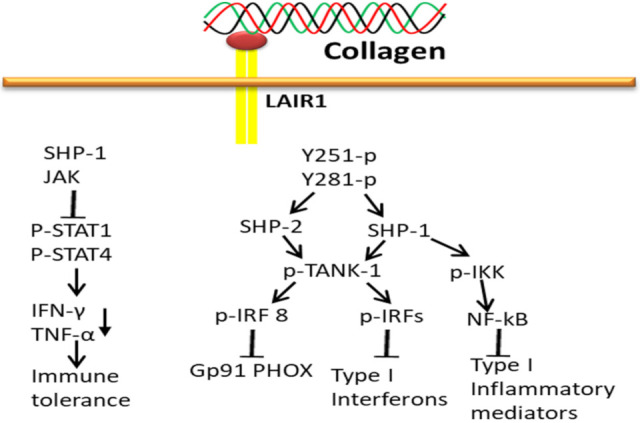
Collagen LAIR1 signals in immune tolerance.

The attachment of collagen to LAIR extracellular surface phosphorylates LAIR1 ITIM tyrosin residues by Src family kinases and further recruits SHP-1 and SHP-2 phosphatases through ITIMs regulatory SH2 domains to carry out phosphatase activity (Figure 10). The activated SHP-1 phosphatase inhibits stimulation and translocation of NF-κB and interferon regulatory factors (IRFs) from the cytoplasm to the nucleus by dephosphorylation of inhibitor of kappa-beta kinase complex (IKK) and preventing TANK-binding kinase 1 (TANK-1) phosphorylation of IRFs, respectively, which subsequently block inflammatory mediators encoding genes transcription. In addition, SHP-2 also blocks phagocyte NADPH oxidase (gp91PHOX) expression by downregulating IRF 8 activation [189].

### 3.6. Collagen-uPARAP/Endo180-Based Signaling Pathways

The uPARAP or Endo180 (CD280 or MRC2)-associated type 1 membrane protein, an endocytic receptor for collagen, belongs to a mannose receptor family consisting of three main domains (a cysteine-rich (CR), fibronectin-type II (FNII) and tandemly arranged C-type lectin-like domains (CTLD, eight in the case of MR)). The collagen-bound uPARAP regulates the collagen turnover in biological cells. However, the actual signaling mechanism of uPARAP with collagen interaction in collagenolysis is not well known. It is established that native intact and partially degraded collagen can be processed by the interaction of uPARAP/Endo180 with components of the plasminogen activator system. The uPARAP interacts with collagen through their common domains such as FNII domain for collagen binding, CR domain for sulfated carbohydrates recognition and CTLD domain for binding to mannose [190].

Therefore, among the three domains, the FnII domain of uPARAP plays a key role in interacting collagen with uPARAP; however, the lack of three domains leads to it being incapable of internalizing collagen [191]. It is opined that long-term culture of uPARAP-/-fibroblasts cells on a reconstituted native collagen I matrix gradually solubilize the collagen through the action of matrix metalloproteases (MMPs). The fragmented collagens are further entered into endocytosis where dissociation occurs in the endosomal compartment to recycle the cell receptor to the cell surface and the collagen ligands are routed further to late endosomes and lysosomes. During endocytosis, the lysosomal enzymes play the main role in the degradation of collagen, especially lysosomal cysteine proteases, which play a prominent part since the specific inhibition of these enzymes leads to lysosomal entrapment and accumulation of internalized collagen [192,193].

Based on the theoretical evidence, most of the diseases are partially (indirectly) or completely (directly) regulated by at least one of the collagen binding cellular receptors such as integrins, DDRs, GPVI, OSCAR, LAIR, Endo180, etc. Additionally, it is opined that the regulation of collagen receptor controls cancer growth, delivery systems and the regeneration of tissues such as skin, bone, tendon, cartilage, neural, etc.

## 4. Conclusions

Overall, this review summarizes the possible signaling mechanism of collagen interacting with the cell receptors for biological functions. The activation of different types of cell receptors highly depends on the molecular pattern and types of collagen. By analyzing the hypothesis, the collagen receptors act as stimulatory and inhibitory receptors for various biological signaling pathways, and the signaling mechanism of collagen-bound receptors is regulated by the microenvironment of cells (intra and extracellular cues). It is evident that regulating specific cell signaling pathways by manipulating the interaction of collagen with its receptor is a breakthrough in future therapeutic treatment. Therefore, this review provides the fundamental basis of the signaling mechanism of collagen-induced cell receptors in normal and diseased physiological functions.

## Data Availability

Not applicable.
